# Supplementary material for: Chlorophyll enhances oxidative stress tolerance in Caenorhabditis elegans and extends its lifespan
Source: PeerJ. 2016 Apr 7;4:e1879. doi: 10.7717/peerj.1879 (PMC4830245; doi:10.7717/peerj.1879)
Supplement: Figure S1 [file peerj-04-1879-s001.pdf]

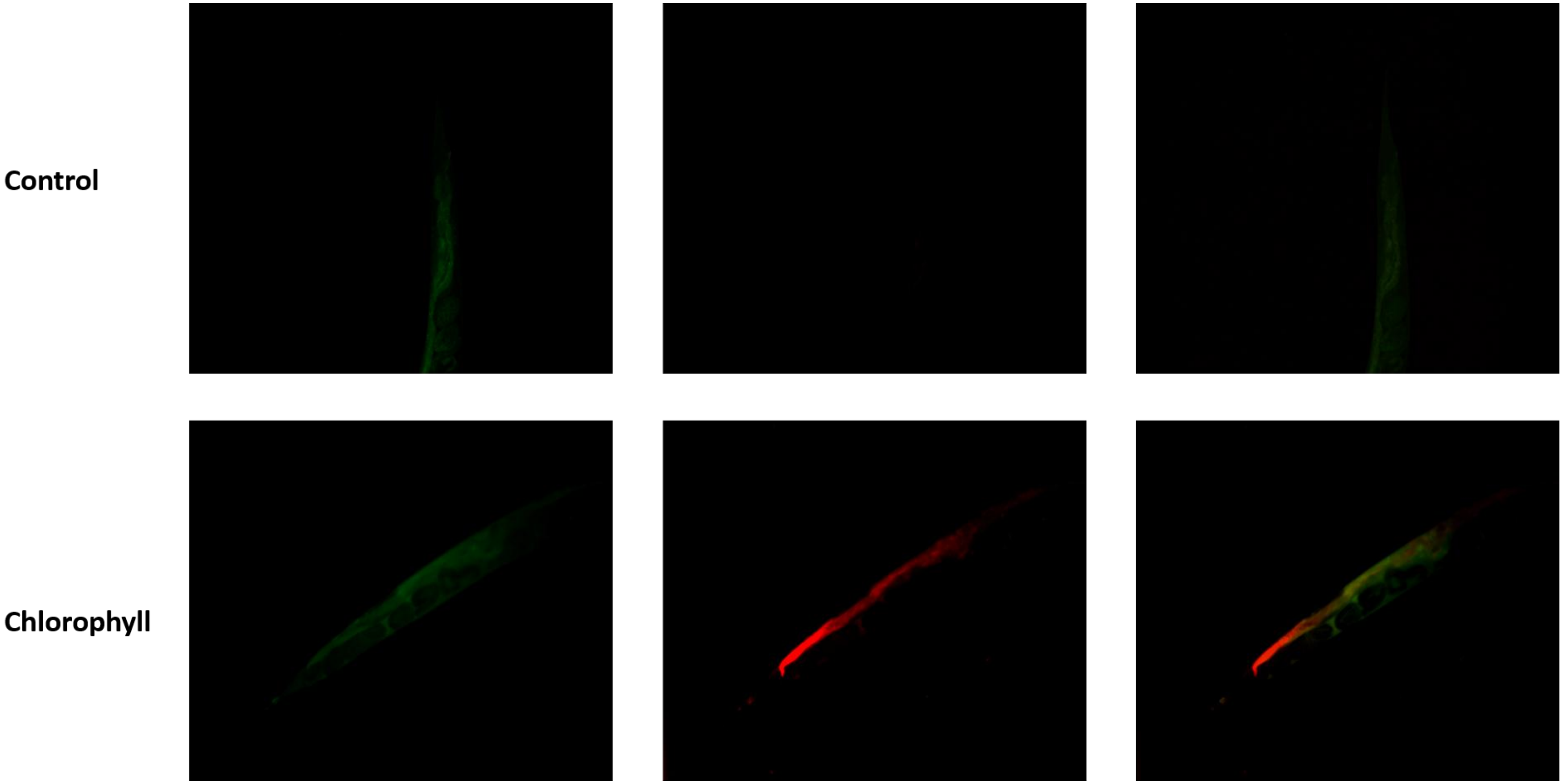

Fig. S 1 Detection of chlorophyll inside the worms. Chlorophyll shows characteristic red fluorescence under the UV-light.
