# Supplementary material for: Chlorophyll enhances oxidative stress tolerance in Caenorhabditis elegans and extends its lifespan
Source: PeerJ. 2016 Apr 7;4:e1879. doi: 10.7717/peerj.1879 (PMC4830245; doi:10.7717/peerj.1879)
Supplement: Data S1 [file peerj-04-1879-s002.pdf]

Raw data for DPPH

**Chlorophyll**				**EGCG**			
Concentration (mg/ml)	Inhibition %			Concentration (µg/ml)	Inhibition %		
0.1	3	3	0	1	19	22	15
1	4	2	12	2	29	34	25
5	3	3	11	3	40	45	37
25	1	1	12	4	50	55	48
100	2	1	7	5	60	66	55
