# Supplementary material for: Chlorophyll enhances oxidative stress tolerance in Caenorhabditis elegans and extends its lifespan
Source: PeerJ. 2016 Apr 7;4:e1879. doi: 10.7717/peerj.1879 (PMC4830245; doi:10.7717/peerj.1879)
Supplement: Data S2 [file peerj-04-1879-s003.pdf]

Raw data of pumping assay

Day 4			Day 5			Day 6			Day 7		
Control	Chlor. 10 mg/ml	Chlor. 50 mg/ml	Control	Chlor. 10 mg/ml	Chlor. 50 mg/ml	Control	Chlor. 10 mg/ml	Chlor. 50 mg/ml	Control	Chlor. 10 mg/ml	Chlor. 50 mg/ml
220	256	279	193	216	223	153	228	195	169	187	210
222	221	246	214	230	228	171	186	218	132	206	193
253	234	235	191	245	263	186	209	216	142	204	182
243	267	228	199	238	240	150	216	208	149	175	192
257	256	249	187	227	234	160	197	215	146	168	185
229	228	269	230	220	203	174	218	228	160	200	189
238	236	246	234	225	210	160	200	213	163	179	180
242	258	250	212	211	266	162	209	214	150	180	205
257	266	274	235	268	240	178	202	223	164	192	197
236	234	269	228	263	265	149	194	217	158	180	188
