# Supplementary material for: Chlorophyll enhances oxidative stress tolerance in Caenorhabditis elegans and extends its lifespan
Source: PeerJ. 2016 Apr 7;4:e1879. doi: 10.7717/peerj.1879 (PMC4830245; doi:10.7717/peerj.1879)
Supplement: Data S3 [file peerj-04-1879-s004.pdf]

Raw data for ABTS

Chlorophyll				Trolox			
Concentration (mg/ml)	Absorbance at 734 nm			Concentration (µg/ml)	Absorbance at 734 nm		
500	0.216	0.214	0.205	10	0.072	0.070	0.074
250	0.308	0.309	0.287	5	0.071	0.070	0.072
125	0.392	0.386	0.375	2.5	0.077	0.074	0.030
62.5	0.437	0.434	0.421	1.25	0.124	0.120	0.129
31.25	0.489	0.487	0.487	0.625	0.377	0.372	0.379
15.625	0.517	0.506	0.495	0.3125	0.506	0.500	0.510
7.8125	0.532	0.534	0.521	0.15625	0.587	0.582	0.589
3.90625	0.543	0.545	0.530	0.078125	0.610	0.613	0.619
1.953125	0.552	0.559	0.547	0.0390625	0.658	0.663	0.653
0.9765625	0.562	0.563	0.554	0.01953125	0.646	0.649	0.641
