# Supplementary material for: Chlorophyll enhances oxidative stress tolerance in Caenorhabditis elegans and extends its lifespan
Source: PeerJ. 2016 Apr 7;4:e1879. doi: 10.7717/peerj.1879 (PMC4830245; doi:10.7717/peerj.1879)
Supplement: Data S4 [file peerj-04-1879-s005.pdf]

Raw data of SOD-3::GFP expression

	Control	Chlorophyll 10 mg/ml
1	110,86	104,83
2	100,96	133,19
3	149,06	145,91
4	79,98	129,75
5	75,77	149,13
6	122,58	116,31
7	153,06	156,94
8	82,72	118,11
9	120,44	111,21
10	73,66	165,15
11	64,21	168,8
12	93,35	115,8
13	66,17	107,65
14	138	170,3
15	50,66	141,36
16	49	109,02
17	119,3	134,25
18	84,81	114,93
19	85,86	96,44
20	149,36	175,18
21	126,93	109,12
22	55,25	129,36
23	115,9	120,44
24	96,8	137,69
25	105,36	112,89
26	67,76	98,79
27	138,84	133,81
28	89,41	101,82
29	55,21	166,43
30	90,55	112,62
31	144,71	105,19
32	80,88	171,74
33	90,3	121,36
34	126,25	164,78
35	136,2	148,05
36	122,45	144,5
37	87,21	128,2
38	113,54	148,26
39	87,93	155,21
40	76,25	132,01
41	110,44	156,78
42	113,43	122,65
43	97,88	119,78
44	74,93	131,56
45	129,43	142,67
46	83,68	109,87
47	97,65	123,42
48	96,36	136,98
49	104,22	148,54
50	98,87	132,58
