# Supplementary material for: Chlorophyll enhances oxidative stress tolerance in Caenorhabditis elegans and extends its lifespan
Source: PeerJ. 2016 Apr 7;4:e1879. doi: 10.7717/peerj.1879 (PMC4830245; doi:10.7717/peerj.1879)
Supplement: Data S5 [file peerj-04-1879-s006.pdf]

# Raw data of quantitation of hsp-16.2/GFP expression

The GFP expression density in the pharynx of TJ375 worms

| | Juglone | Juglone+Chlorophyll |
|---|---|---|
| 1 | 36.215 | 15.501 |
| 2 | 14.331 | 21.314 |
| 3 | 38.765 | 29.118 |
| 4 | 42.545 | 14.696 |
| 5 | 40.975 | 34.855 |
| 6 | 45.744 | 16.997 |
| 7 | 34.855 | 13.983 |
| 8 | 35.68 | 11.206 |
| 9 | 74.531 | 14.944 |
| 10 | 83.567 | 20.017 |
| 11 | 28.574 | 12.61 |
| 12 | 44.767 | 20.97 |
| 13 | 71.951 | 9.474 |
| 14 | 48.279 | 8.197 |
| 15 | 60.87 | 41.862 |
| 16 | 35.023 | 46.834 |
| 17 | 18.301 | 51.313 |
| 18 | 9.598 | 13.388 |
| 19 | 59.237 | 22.775 |
| 20 | 54.26 | 18.675 |
| 21 | 28.605 | 27.173 |
| 22 | 40.217 | 13.115 |
| 23 | 78.374 | 11.525 |
| 24 | 61.638 | 19.282 |
| 25 | 53.874 | 13.386 |
| 26 | 59.73 | 17.925 |
| 27 | 68.191 | 59.2 |
| 28 | 56.042 | 43.486 |
| 29 | 53.667 | 52.999 |
| 30 | 56.514 | 37.047 |
| 31 | 47.462 | 44.538 |
| 32 | 40.584 | 55.413 |
| 33 | 39.8 | 37.891 |
| 34 | 44.187 | 56.403 |
| 35 | 45.061 | 14.19 |
| 36 | 27.148 | 14.667 |
| 37 | 46.071 | 17.17 |
| 38 | 46.672 | 57.409 |
| 39 | 31.405 | 20.325 |
| 40 | 43.418 | 24.62 |
| 41 | 50.074 | 51.57 |
| 42 | 29.98 | 43.797 |
| 43 | 43.61 | 49.348 |
| 44 | 41.073 | 19.254 |
| 45 | 30.283 | 38.303 |
| 46 | 34.047 | 17.004 |
| 47 | 42.708 | 14.242 |
| 48 | 45.92 | 14.558 |
| 49 | 48.649 | 12.865 |
| 50 | 35.398 | 10.453 |
